# Is Immediate Processing of Presupposition Triggers Automatic or Capacity-Limited? A Combination of the PRP Approach with a Self-Paced Reading Task

**DOI:** 10.1007/s10936-019-09686-3

**Published:** 2020-02-06

**Authors:** Cosima Schneider, Nadine Bade, Markus Janczyk

**Affiliations:** 1 Department of Psychology, University of Tübingen, Schleichstraße 4, 72076 Tübingen, Germany; 2 Department of Cognitive Studies, École Normale Supérieure, Paris, France; 3 Department of Psychology, University of Bremen, Bremen, Germany

**Keywords:** Presuppositions, Experimental pragmatics, Dual-task, PRP

## Abstract

Informally speaking, presuppositions are meaning components which are part of the common ground for speakers in a conversation, that is, background information which is taken for granted by interlocutors. The current literature suggests an immediate processing of presuppositions, starting directly on the word triggering the presupposition. In the present paper, we focused on two presupposition triggers in German, the definite determiner *the* (German *der*) and the iterative particle *again* (German *wieder*). Experiment 1 replicates the immediate effects which were previously observed in a self-paced reading study. Experiment 2 then investigates whether this immediate processing of presuppositions is automatic or capacity-limited by employing the psychological refractory period approach and the locus of slack-logic, which have been successfully employed for this reason in various fields of cognitive psychology. The results argue against automatic processing, but rather suggest that the immediate processing of presuppositions is capacity-limited. This potentially helps specifying the nature of the involved processes; for example, a memory search for a potential referent.

## Introduction

Language and communication are ubiquitous in everyday life and speakers often communicate more than they actually say. How this additional meaning arises is an important question in the study of natural language meaning. Presuppositions are an example of meaning components that can be distinguished from the purely asserted meaning of an utterance, and have been a vital topic in the semantic and pragmatic literature of the last decades (see Beaver and Geurts 2012). While much of the previous work on presupposition processing focused on the influence of different contexts on the interpretation of presuppositions, the main goal of the present paper is to investigate at which stage of cognitive processing presuppositions unfold their impact.

### Presuppositions and Their Immediate Processing

From a theoretical point of view, the term *presupposition* refers to background information which is taken for granted by speaker and listener. It differs from the *assertion* of a sentence, which is novel content and part of the main meaning of an utterance. Presuppositions are modeled as restrictions on what are appropriate contexts for the utterance (Heim 1991; Heim and Kratzer 1998; Stalnaker 1973), that is, propositions that must be entailed by the context in order for a sentence with a presupposition to be felicitously uttered and added to the common ground (Heim 1990). The context (set) or common ground is defined as the set of propositions believed to be true by all participants of a conversation. More formally speaking, a sentence *p* presupposes *q* if the use of *p* is inappropriate when *q* is not in the common ground (Stalnaker 2002). Under a semantic view, certain linguistic expressions trigger these appropriateness conditions and are therefore called *presupposition triggers*. In (1), for example, the word *again* triggers the presupposition that Anna has already scored before yesterday.

1. Yesterday, Anna scored again.

As a result, a sentence as in (1) is predicted to only be appropriate (i.e., felicitous) in contexts which entail that Anna scored before. If the context does not entail this information, the sentence is predicted to be infelicitous. There is, however, a rescue strategy for sentences like (1) if the presupposition is not fulfilled. So-called *accommodation* describes the process of just assuming the presupposition to hold on the part of the speaker. It has been observed that accommodation is a highly context-dependent process (based on the probability of the truth of the presupposition in the given context; Heim 1992). For example, (1) might be surprising given that Anna never plays soccer. However, if she is known to be a very talented striker it is quite unsurprising. It has also been claimed that the availability of accommodation is dependent on the type of trigger and more difficult for triggers like *again* (see more discussion below).

Presuppositions are differentiated from asserted meaning and conversational implicatures, because they have different properties. For example, unlike assertions, presuppositions survive embedding under certain operators such as negation, conditionals, modals, or questions. The sentence in (2), for example, still presupposes that Anna scored again. However, it does not assert anymore that she scored yesterday.

(2) If Anna scored again yesterday, I’d be surprised.

As pointed out above, presuppositions are assumed to be encoded in a lexical trigger according to a semantic view, that is, they are associated with certain words (Frege 1892; Heim 1982; Russell 1905). This view is in line with the prediction that the trigger itself leads to awareness of the importance of context and could thus evoke immediate processing costs. However, there is an alternative theoretical perspective on presuppositions which takes a more pragmatic approach (Stalnaker 1973; Levinson 1983; Simons 2001). It assumes that presuppositions are not semantically encoded but are pragmatic, that is, they only play a role after the sentence’s main meaning is computed and its integration into the context is considered. This “two-step” procedure means that the presupposition is not necessarily processed immediately, but only later at the end of the sentence. How presuppositions arise is still a highly debated issue in the literature (“the triggering problem”). It led to the debate whether presuppositions are needed as a separate concept or whether the issue is better understood in terms of what is at-issue or raises attention versus what is non-at-issue/in the background (Simons et al. 2011; Abrusán 2011; Tonhauser et al. 2018).^1^ So far, there is a lot of evidence supporting the view that presuppositions are processed immediately (see below), which speaks against a two-step process. The data thus suggest that any processing model of presuppositions should contain the trigger itself as an important factor.

Experimental evidence for immediate processing of presuppositions comes from various methods. For example, Kirsten et al. (2014) investigated the processing of presuppositions while measuring event related potentials (ERPs) of the EEG in an experiment focusing on the presuppositions triggered by the definite determiner, compared to inferences arising from the indefinite determiner. Participants were presented with test sentences word-by-word on a computer screen and were asked comprehension questions at the end of the experiment. The data revealed ERP effects already on the trigger word. This led the authors to conclude that presupposition processing begins as soon as the presupposition trigger is encountered. Burkhardt’s (2006) ERP study further supports the idea of early processing of presuppositions by revealing an N400 effect on the trigger position when the existence presupposition of the definite determiner was not given. The experiment varied the degree of availability of referents for definite determiner phrases by manipulating the context (*given*, *bridged*, and *new*). Definite noun phrases that were completely novel elicited N400 and P600 components compared to definite noun phrases whose referents were given in the context. In cases where the referent could easily be inferred (e.g., “the bus driver” in situations describing somebody entering a bus), the effect was weaker. In a follow-up study, Burkhardt (2007) manipulated the terms of inferential demands needed to form a relationship between the definite noun phrase and the information of the context sentence, which was previously presented. It was either necessary or inducible information. Drawing more demanding inferences resulted in larger P600 effects, whereas no N400 effects were observed when the context did not support the presupposition. Jouravlev et al. (2016) also examined ERPs, but focused on the PSP trigger *again* (in English). Participants read sentences in contexts that either supported the presupposition (e.g., “Jake had tipped a maid at the hotel once before. Today he tipped a maid at the hotel again…”) or violated it (e.g., “Jake had never tipped a maid at the hotel before. Today he tipped a maid at the hotel again…”). The data analysis revealed the expected effects for semantic and syntactic violations (N440 and P600). Summing up, these results provide evidence for a rapid, on-line integration of presupposed content triggered by the adverb *again*. However, the observed pattern differs from the pattern reported for definite determiners.

Domaneschi et al. (2018) also investigated presupposition processing in different contextual conditions. To this end, they used contexts that satisfied the presupposition versus contexts that were neutral with regard to the truth of the presupposition (i.e., required accommodation), and compared two types of triggers, that is, definite descriptions and change-of-state verbs. The results also support the idea of immediate presupposition processing in the accommodation condition (a biphasic N400–P600 pattern at the point where the presupposition is known), but furthermore show that the two triggers differ in processing: for definite descriptions, a clear involvement of the N400 was observed, while for change-of-state verbs the costs of accommodation were associated with a more pronounced P600. The data support the idea that presupposition accommodation involves two steps: (1) search for a previous antecedent in the discourse, and in case of an unsuccessful search, (2) a second step of context repairment, namely an integration of the presupposed content into the discourse model.

In sum, these EEG studies provide evidence for an immediate processing of presuppositions, starting on the trigger itself. It is important to note that all of the studies presented focused on the influence of context, that is, they compared the processing cost of accommodation with the processing costs of a satisfied presupposition. In contrast, the present study focused on comparing a presupposition trigger with non-trigger words, and on the question whether processing the trigger is a capacity-limited process.

Other studies on presupposition processing used reading times. For example, Schwarz (2007) focused on the German additive particle and presupposition trigger *auch* (Engl. *too*) and reported longer reading times for clauses containing the trigger *auch* when the presupposition was not satisfied compared to when it was. Of particular importance for the present purposes is Experiment 1 of Tiemann et al. (2011). These authors also employed self-paced reading to investigate at which point in time processing of presuppositions takes place and included five different presupposition triggers (German *wieder*, Engl. *again*; *auch*, Engl. *also*; *aufhören*, Engl. *stop*; *wissen*, Engl. *know*; and definites in the shape of possessive noun phrases [*sein/ihr,* Engl. *his/her*]). In their experiment, they compared (1) sentences with a presupposition trigger, (2) grammatical sentences without a trigger, and (3) ungrammatical sentences without a trigger. The sentences were presented in contexts which did not explicitly verify the presupposition (i.e., they were neutral with regard to the presupposition). Overall, reading times at the positions of the trigger and the following word were longest in sentences with presupposition triggers, intermediate in grammatical sentences, and shortest in the ungrammatical sentences. These effects also indicate that a presupposition trigger is considered immediately upon encountering it. However, recent studies suggest that different types of presupposition triggers differ in processing (Abrusán 2011; Domaneschi et al. 2014; Domaneschi et al. 2018; Domaneschi and Di Paola 2018; Jouravlev et al. 2016; Tiemann et al. 2015). Against this background, it is unfortunate that Tiemann et al. (2011) did not analyze reading times for the different triggers separately. It thus remains unclear whether the results are similar for all triggers or just for a subset of them.

In sum, the current literature suggests an immediate processing of presuppositions, which starts directly on the trigger. The present study goes a step further by asking whether this immediate processing is automatic or capacity-limited. More precisely, we investigated this for two selected triggers, definite determiners and *again*, using a similar methodology as Tiemann et al. (2011, Exp. 1). The choice of triggers is partly motivated by the theoretical discussion in Kripke (2009), who argued that presuppositions triggered by *again* and *too* are especially hard to accommodate compared to definite determiners. The choice is also motivated by the classifications that were suggested to account for differences in processing. More specifically, Tiemann et al. (2015) suggested to categorize the triggers *again* and definite determiner in two different classes based on their different behavior. They proposed a maxim of interpretation which they called *Minimize Accommodation*: “Do not accommodate a presupposition unless missing accommodation will lead to uninterpretability of the assertion.” According to this classification, Class 1 comprises triggers that are likely to be ignored in case of presupposition failure (e.g., particles like *again*, *too*, and *even*), because their presuppositions are not relevant to the assertion (and can thus be ignored given *Minimize Accommodation*). On the other hand, presuppositions of triggers in Class 2 must be accommodated according to this view, because otherwise the utterance cannot be interpreted (e.g., definite descriptions, factives, and change of state verbs), as these triggers do contribute to the assertion (see also Glanzberg 2005, for a similar distinction). Processing of presuppositions associated with the definite determiner and *again* should be different following this proposal: *again*, being a Class 1 trigger, does not contribute anything to the assertion of the sentence. That is, the sentence in (3) can be evaluated with regard to its truth conditional content (that Jenna went ice-skating) without knowing the presupposition. This is not the case for triggers belonging to Class 2 such as, for example, definite determiners. The truth of the sentence in (4) cannot be evaluated without the presupposition of existence and uniqueness being verified, that is, without knowing whether there is a sun and whether it is unique.

(3) Jenna went ice-skating, again.
(4) The sun is shining.

We therefore focus on these two triggers, which have been argued to belong to different categories. Focusing on only two triggers has the advantage that we will be able to increase the number of stimuli per participant to allow for meaningful separate analyses of the two triggers.

### The Locus of Slack-Logic and an Example Application

To determine whether presupposition processing is automatic or a capacity-limited process, we will use the psychological refractory period (PRP) approach, a method that has been widely used in cognitive psychology with its origin in dual-task research. Of particular importance is the locus of slack-logic (Schweickert 1978) within a PRP experiment. We will introduce the general logic with an experimental example in the following, and will adapt this logic to a self-paced reading task.

**Fig. 1:**
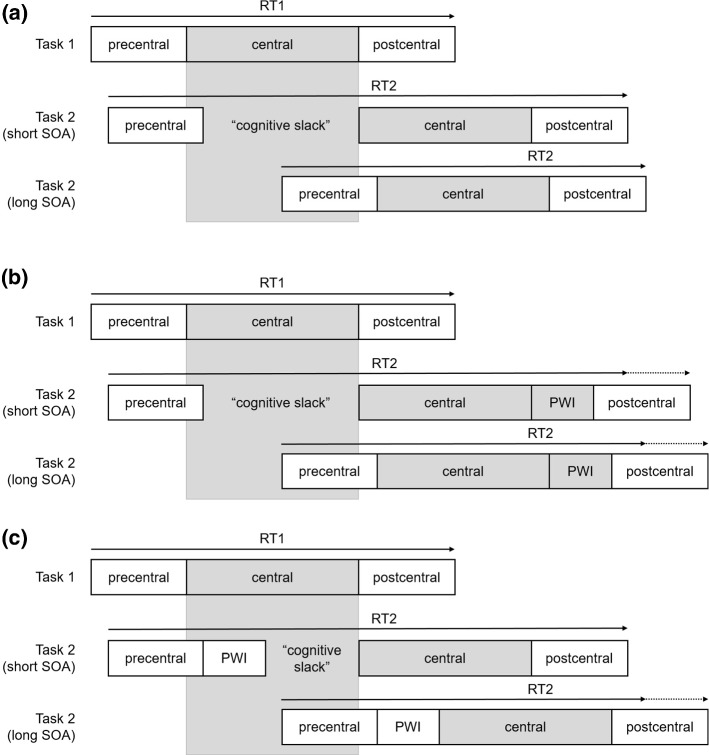
Illustration of the central bottleneck model (**a**) and the predictions of the locus of slack-logic (**b**, **c**). (Note: *PWI* picture word interference)

In general, participants perform two independent tasks in each trial of a PRP experiment. The critical manipulation is the stimulus onset asynchrony (SOA), which is the time between the presentation of the Task 1 stimulus (S1) and the Task 2 stimulus (S2). With a short SOA, the two tasks overlap temporally, whereas there is no or only little temporal overlap with long SOAs. The typically observed result pattern is that the response time in Task 1 (RT1) does not depend on SOAs, but those in Task 2 (RT2) become longer the shorter the SOA—the PRP effect (Telford 1931). The most widely accepted explanation for this observation is the central bottleneck model (e.g., Pashler 1994; Welford 1952; see Fig. 1a for an illustration). A starting assumption of this model is that processing of a task is split into three stages: (a) a precentral stage, (b) a central stage, and (c) a postcentral stage. The precentral stage has most often been related to (early) perceptual processing and the postcentral stage to motor processing and execution. It is assumed that these two stages can run in parallel with all other stages of simultaneously processed tasks. The central stage has originally been related to response selection (Pashler 1994), but other processes seem to require this stage as well, for example, encoding into short-term memory (Jolicoeur and Dell’Acqua 1998), selection of working memory items (Janczyk 2017), or anticipation of action effects (Wirth et al. 2015; see Janczyk and Kunde, under review). In contrast to the two other stages, the central stage is conceived as capacity-limited and can only be invoked by one task at a time, thereby constituting a bottleneck. With a short SOA, the central stage of Task 1 is not yet processed when the precentral stage of Task 2 has finished. Thus, central processing of Task 2 has to wait until the bottleneck is available again. This time of waiting is called the *cognitive slack* and is what leads to long RT2s with a short SOA. With a long SOA, in contrast, no cognitive slack occurs and Task 2 processing is not interrupted, resulting in short RT2s.

Importantly, this model can also be used to distinguish at which stage of processing a particular RT effect emerges (i.e., its “locus” in processing), and by implication then, whether this process is automatic or capacity-limited. We will explain this with a study by Piai et al. (2014) as an example, who investigated the locus of semantic interference in picture-word interference (PWI) experiments (see Abdel Rahman and Melinger 2009). Typically, participants are presented with pictured objects and distractor words and are instructed to name the picture while ignoring the distractor word. Naming latencies are shorter when picture and word match than when they do not. Piai et al. asked whether this semantic interference effect arises during the precentral stage, and thus is the result of parallel processing (Dell’Acqua et al. 2007), or during the capacity-limited central stage that was related to lexical selection (Schnur and Martin 2012). To illustrate, consider Piai et al.’s Experiment 1.^2^ Task 1 was to give a manual response to a low- or high-pitched tone, and Task 2 was a vocal naming response to a picture combined with a distractor word. Pictures of the body parts leg, arm, and finger were combined with the corresponding word or a string of five Xs. In congruent trials, pictures and words matched, in incongruent trials, they did not match. In neutral trials, the pictures were presented with the five Xs. The SOA between the tone and the PWI stimulus was either 0 or 500 ms.

Two different predictions can be derived from the central bottleneck model. First, consider that the PWI effect results from processing during the capacity-limited central stage (see Fig. 1b). With a long SOA, Task 2 RTs are prolonged in incongruent compared to congruent trials (visualized by the gray box labeled PWI). Because with a short SOA the central stage can only start after the central stage of Task 1 has finished, the same PWI effect is expected in this case. In other words, the PWI effect is expected to combine additively with SOA. Second, consider that the PWI effect emerges from parallel processing that can run simultaneously with the central stage of Task 1 (see Fig. 1c). With the long SOA, the same prediction as for the previous case is made and the PWI effect should be observed. With a short SOA, in contrast, the processing leading to the PWI effects starts regardless of the central stage of Task 1, and any additional processing required in incongruent trials stretches into the cognitive slack. As a consequence, the PWI effect becomes invisible at the short SOA and SOA and PWI are expected to produce an (underadditive) interaction. The data clearly revealed an additive effect of SOA and PWI what suggests that the PWI effect requires central capacity and arises during (or after) the central stage. The results of further experiments in Piai et al. (2014) support this, because the additivity robustly replicated across these other experiments.

### The Present Study: Is Processing of Presupposition Triggers Capacity-Limited or Automatic?

In the present study, we will use the PRP approach we just introduced to investigate the processing of presuppositions triggered by *again* and by definite determiners in more detail. The major question of our study is whether processing initiated when encountering a presupposition trigger is automatic or requires limited capacities. Experiment 1 was designed after Experiment 1 of Tiemann et al. (2011) with several goals. First, we aimed at replicating the observation of longer reading times for triggers compared with neutral or unacceptable sentences (Tiemann et al. 2011; see also Schwarz 2007, for the trigger *auch* compared to the neutral word *vorher* [Engl. *earlier*]). Second, because we needed to use a slightly modified presentation method of the words in the self-paced reading task to apply the PRP setup and the locus of slack-logic in Experiment 2, we already adopted this method in Experiment 1 to ensure that the longer reading times for triggers are also observed under these conditions. Third, based on the acceptability ratings of sentences collected in Experiment 1, we selected those items that fit best for use in the subsequent experiment. In Experiment 2, we then adapted the PRP approach to the reading task by adding a tone discrimination task and presenting the trigger (or the corresponding word at this position) after a variable SOA following the tone. To ensure that participants interpreted the sentences in the intended way, we again included the rating after each trial and asked comprehension questions at the end of the experiment. We would like to stress at this point that conclusions about differences between the triggers can only be made if the qualitative pattern we observe is different. Numerical differences, even if substantiated by significant main effects, do not necessarily mean that the underlying processes are different. For example, the processes may simply require more time because they are more difficult in one condition.

## Experiment 1

Experiment 1 uses a self-paced reading task to investigate and establish the reading times for several regions of interest (i.e., the presupposition trigger, the word following the presupposition trigger, the final word, and the total reading time) separately for two particular presupposition triggers, namely determiners and the German word *wieder* (Engl. *again*). Additionally, this experiment prepared the subsequent Experiment 2, which focuses on the main question of this paper. To this end, participants rated acceptability of sentences against the presented context after each trial. On the basis of these data, we selected the sentences for the following experiment. Furthermore, Experiment 2 required the simultaneous presentation of all words preceding the presupposition trigger or the corresponding word on the trigger position to apply the locus of slack-logic. Thus, we already used this procedure in Experiment 1 to determine whether or not we still observe an effect of the presupposition trigger in reading times.

Although this experiment is closely designed after Experiment 1 of Tiemann et al. (2011), we used only two triggers as opposed to the five different triggers used by Tiemann et al. This allowed us to increase the number of times each trigger was presented in the experiment. Following Tiemann et al., we will first visualize reading times averaged for both triggers, but—if warranted—this is followed-up by analyses of both triggers separately. By and large, the expectation was to replicate the results obtained by Tiemann et al. despite the changes in the presentation procedure and to identify possible differences between the two triggers belonging to different categories.

### Method

#### Participants

Forty-eight native speakers of German (35 female, 13 male; mean age = 24.4 years) participated in this experiment. They were recruited from the participant pool at the University of Tübingen (Germany), were naïve regarding the hypotheses of this experiment, and signed informed consent prior to data collection. Participants received 8€ or course credit for their participation.

#### Apparatus and Stimuli

Stimulus presentation and response collection were controlled by a standard PC connected to a 17-in. CRT monitor. Responses in the reading task were given on an external response key which was located to the right of the participants and was operated with the right index-finger. Ratings of the sentences were provided via the number keys 1–4 on a standard QWERTZ keyboard ranging from very unnatural (1) to very natural (4).

**Fig. 2:**
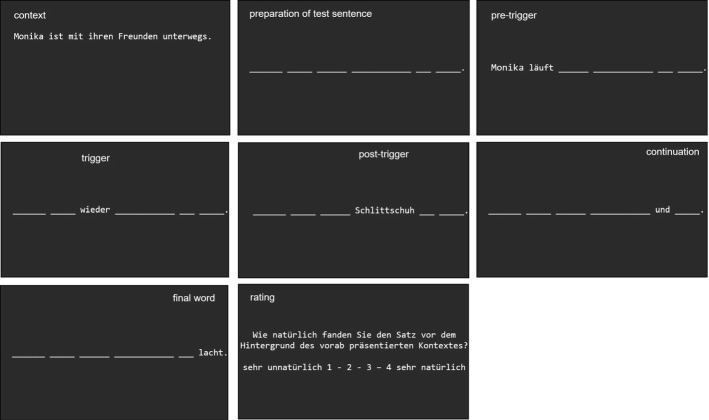
Illustration of the task used in Experiment 1 (see text for more information). (Note that the words appearing in the upper part (“context”, “preparation of test sentence”, …) did not actually appear during the experiment but were added here for clarity)

All stimuli were presented in white font on a black background. Context sentences were presented in full length in the upper half of the screen. The letters of the test sentences’ words were first substituted by underscores as placeholders. All words preceding the presupposition trigger or the corresponding word on this position were presented simultaneously; all subsequent words were presented one-by-one (see below, section “Task and Procedure” for more information). Once a new word was presented, the previous word disappeared and was again substituted with the underscores (see Fig. 2).

We included two types of presupposition triggers in this experiment, namely the German definite determiner *der* (Engl. *the*) and the German iterative particle *wieder* (Engl. *again*). For each trigger, we created 52 sets of experimental sentences, thus 104 sets in total. Each set consisted of a context sentence and three test sentences. The context sentences merely introduced the protagonists, but were kept as neutral as possible with regard to the truth of the presupposition. They were designed so that they made the acceptable test sentence appropriate, the trigger sentence somewhat degraded due to the presupposition being neither true nor false in the context, and the unacceptable sentence inappropriate [see (5) and (7)]. The test sentences contained either a presupposition trigger [(6a) and (8a)], a neutral word [(6b) and (8b)], or a semantically unacceptable word [(6c) and (8c)]. The neutral/unacceptable words replaced the trigger word in the respective conditions and kept the sentence semantically acceptable or made it semantically unacceptable. In total, 312 trials resulted.

Example item *again*

(5) Kontext: Monika ist mit ihren Freunden unterwegs.
  Context: Monika is with her friends out.

(6) Test sentences:
  - Monika läuft**wieder** Schlittschuh und lacht. (trigger)
    Monika does**again** ice-skating and smiles.
  (b) Monika läuft**heute** Schlittschuh und lacht. (neutral)
    Monika does**today** ice-skating and smiles.
  (c) Monika läuft**freundlich** Schlittschuh und lacht. (unacceptable)
    Monika does**friendly** ice-skating and smiles.

Example item *determiner*

(7) Kontext: Marie sonnt sich heute im Garten.
  Context: Marie suns herself today in (the) garden.

(8) Test sentences:
  - Marie liegt auf**der** Liege und trinkt Wasser. (trigger)
    Marie lies on**the** lounger and drinks water.
  (b) Marie liegt auf**einer** Liege und trinkt Wasser. (neutral)
    Marie lies on**a** lounger and drinks water.
  (c) Marie liegt auf**jeder** Liege und trinkt Wasser. (unacceptable)
    Marie lies on**every** lounger and drinks water.

When creating context and test sentences, we pursued the same goals as Tiemann et al. (2011) did. Most importantly, we made the sentences as neutral as possible with regard to the presupposition, that is, they did not explicitly verify or falsify it. At the same time, we made the events described plausible in the given setting so that the “neutral” test condition would be completely acceptable, the trigger sentence somewhat acceptable (requiring accommodation, however), and the unacceptable sentence the most unacceptable (as it was ill-formed irrespective of plausibility in the context).

#### Task and Procedure

Each trial started with the complete context sentence, horizontally centered in the upper part of the computer screen (see Fig. 2 for an illustration of the following). After participants read the sentence, they were to press the response button to request the test sentence. The test sentence was presented in a self-paced reading manner. This allows readers to use the response button presses to control the exposure duration for each section of the sentence they read. The test sentence was divided into a segment preceding the trigger word or the corresponding word on this position [the underlined part in Examples (6) and (8)], in which all words were presented simultaneously, and a section following it. Since simultaneous presentation applied to all sentence types, it was up to then equally likely for a participant to be confronted with a trigger sentence, a neutral sentence, or an unacceptable sentence. The following words, that is the presupposition trigger itself, the neutral word, or the unacceptable word [printed in bold font in Examples (6) and (8)], and all subsequent words were presented word-by-word upon response key presses. Reading times were measured from word/segment onset until the response key was pressed. After the test sentence was read, participants rated the acceptability of the test sentence within the given context.

Participants started with reading written instructions. This was followed by a short practice block with two sets of each trigger in all three conditions, thus 12 trials in total. The order of these practice trials was determined randomly, but was the same for all participants. Then, the 300 test trials were administered in three blocks of 100 trials each. The order of presentation was random, with the restriction that sentences of the same item did not appear in different conditions directly in succession. All participants were tested individually in a single session of about 60 minutes. This is another slight change compared to the original study: Tiemann et al. (2011) tested participants in three separate sessions to avoid that they saw the same item in different conditions within one session. As we increased the number of stimuli though, we did not expect recognition effects during one session.

#### Design and Analyses

The independent variables of interest were (1) sentence type (trigger vs. neutral vs. unacceptable) and (2) trigger type (determiner vs. *again*). Mean acceptability ratings were submitted to a 3 × 2 Analysis of Variance (ANOVA) with sentence type and trigger type as repeated-measures. Reading times were calculated per letter (see Tiemann et al. 2011) for the following regions: (1) the word(s) preceding the trigger position (pre-trigger), (2) the presupposition trigger or the corresponding word on this position (trigger), (3) the word following the trigger position (post-trigger), the final word (final word), and the reading time of the whole sentence (total). Trials in which one reading time deviated more than 2.5 standard deviations from the respective design cell (calculated separately for each participant) were excluded as outliers (11.01% of the trials). Mean reading times for each region were submitted to the same ANOVA as acceptability ratings were. When the interaction of trigger type × sentence type was significant, we ran separate ANOVAs for both triggers with sentence type as a repeated-measure. A significant main effect in this analysis was followed up by paired *t* tests. In case of violations of the sphericity assumption, uncorrected degrees of freedom are reported, but the corresponding ε-estimate is provided. Effect sizes for *t* tests were calculated as $d = \frac{t}{\sqrt n }$ with *n* = 48.

### Results

#### Acceptability Rating

**Fig. 3:**
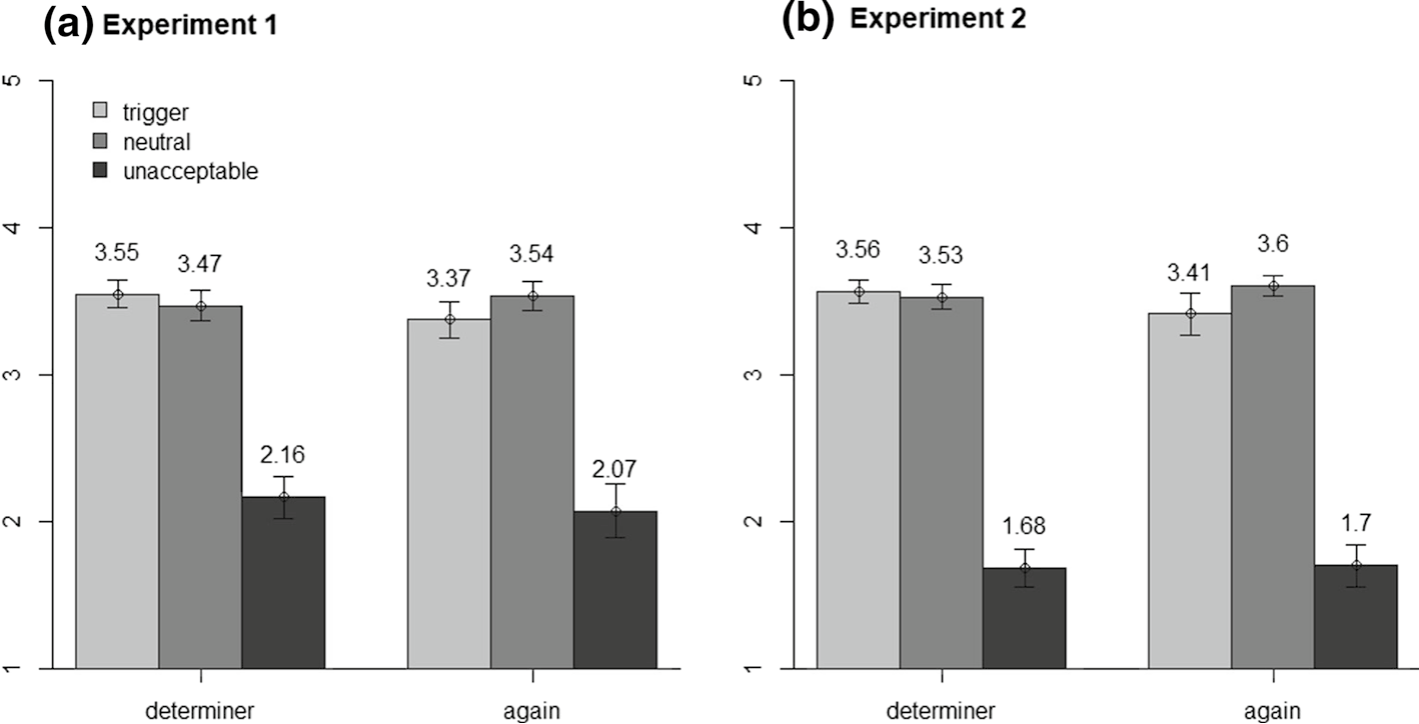
Acceptability ratings in Experiments 1 and 2 as a function of sentence type and trigger type. Error bars are 95% confidence intervals for the means

Results of the acceptability rating are visualized in Fig. 3a. Unacceptable sentences were rated worst and trigger and neutral sentences were rated much more appropriate. Descriptively, for the determiner condition, ratings for neutral sentences were slightly worse than for trigger sentences, whereas for the trigger *again*, neutral sentences were rated best. The ANOVA revealed a main effect of sentences type, *F*(2,94) = 330.60, *p* < .001, η_p_^2^ = .88, ε = .55, and of trigger type, *F*(1,47) = 4.78, *p* = .034, η_p_^2^ = .09. The interaction was significant as well, *F*(2,94) = 6.08, *p* = .007, η_p_^2^ = .11, ε = .77, and we therefore analyzed the two triggers separately.

For the determiner condition, the ANOVA revealed a main effect of sentence type *F*(2,94) = 302.87, *p* < .001, η_p_^2^ = .87, ε = .59. Significant differences were obtained between all sentence types, trigger versus neutral: *t*(47) = 3.10, *p* = .003, *d* = 0.45; unacceptable versus trigger: *t*(47) = 17.89, *p* < .001, *d* = 2.58; unacceptable versus neutral: *t*(47) = 17.89, *p* < .001, *d* = 2.58. For the trigger *again*, the main effect of sentence type was significant as well, *F*(2,94) = 213.66, *p* < .001, η_p_^2^ = .82, ε = .61, and the *t* tests revealed significant differences between all sentence types, trigger versus neutral: *t*(47) = 4.58, *p* < .001, *d* = 0.66; trigger versus unacceptable: *t*(47) = 14.62, *p* < .001, *d* = 2.11; neutral versus unacceptable: *t*(47) = 15.53, *p* < .001, *d* = 2.24.

#### Reading Times

**Fig. 4:**
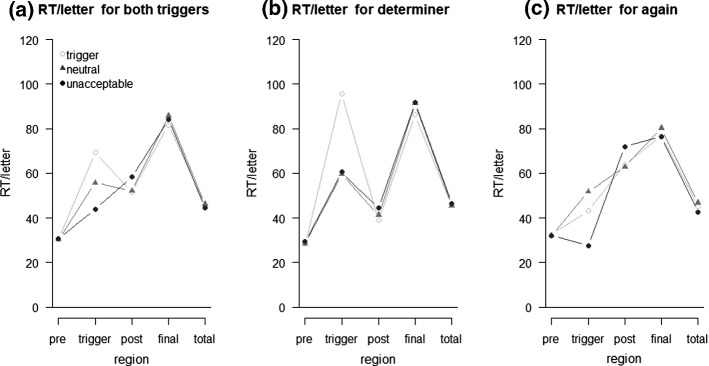
Reading times (RT; in milliseconds) per letter of Experiment 1 analyzed across triggers in (**a**), and separately for the two triggers determiner (**b**) and *again* (**c**) for the regions pre-trigger (pre), trigger, post-trigger (post), final word (final), and total as a function of sentence type

Reading times per letter across both triggers are visualized in Fig. 4a, and separately for the determiner and *again* in Fig. 4b and c, respectively. All inferential statistics are summarized in Table 1. The ANOVA revealed significant differences between the two trigger types for all analyzed positions, perhaps pointing to differences in how the two triggers are processed.

**Table 1:** Inferential statistics for Experiment 1

	Pre-trigger	Trigger	Post-trigger	Final word	Total
Trigger type	*F*(1,47) = 82.37, *p* < .001, η_p_^2^ = .64	*F*(1,47) = 628.80, *p* < .001, η_p_^2^ = .93	*F*(1,47) = 472.96, *p* < .001, η_p_^2^ = .91	*F*(1,47) = 39.87, *p* < .001, η_p_^2^ = .46	*F*(1,47) = 6.69, *p* = .013, η_p_^2^ = .12
Sentence type	*F*(2,94) = 0.86, *p* = .426, η_p_^2^ = .02	*F*(2,94) = 544.50, *p* < .001, η_p_^2^ = .92, ε = .73	*F*(2,94) = 25.23, *p* < .001, η_p_^2^ = .35, ε = .60	*F*(2,94) = 0.73, *p* = .439, η_p_^2^ = .02, ε = .69	*F*(2,94) = 3.01, *p* = .073, η_p_^2^ = .06, ε = .71
Interaction	*F*(2,94) = 2.99, *p* = .055, η_p_^2^ = .06	*F*(2,94) = 409.19, *p* < .001, η_p_^2^ = .90, ε = .88	*F*(2,94) = 10.67, *p* < .001, η_p_^2^ = .19, ε = .87	*F*(2,94) = 1.11, *p* = .334, η_p_^2^ = .02	*F*(2,94) = 22.54, *p* < .001, η_p_^2^ = .32, ε = .87
Only determiner					
Sentence type		*F*(2,94) = 466.72, *p* < .001, η_p_^2^ = .91, ε = .86	*F*(2,94) = 8.30, *p* = .003, η_p_^2^ = .15, ε = .63		*F*(2,94) = 0.58, *p* = .530, η_p_^2^ = .01, ε = .83
Trigger versus neutral		*t*(47) = 25.10, *p* < .001, *d* = 3.62	*t*(47) = 3.46, *p* = .001, *d* = 0.50		
Trigger versus unacceptable		*t*(47) = 23.26, *p* < .001, *d* = 3.36	*t*(47) = 3.27, *p* = .002, *d* = 0.47		
Neutral versus unacceptable		*t*(47) = 0.81, *p* = .422, *d* = 0.12	*t*(47) = 2.09, *p* = .042, *d* = 0.30		
Only *again*					
Sentence type		*F*(2,94) = 502.61, *p* < .001, η_p_^2^ = .91, ε = .76	*F*(2,94) = 37.73, *p* < .001, η_p_^2^ = .45, ε = .73		*F*(2,94) = 15.37, *p* < .001, η_p_^2^ = .25, ε = .71
Trigger versus neutral		*t*(47) = 13.60, *p* < .001, *d* = 1.96	*t*(47) = 1.58, *p* = .121, *d* = 0.23		*t*(47) = 3.53, *p* = .001, *d* = 0.51
Trigger versus unacceptable		*t*(47) = 23.01, *p* < .001, *d* = 3.32	*t*(47) = 6.04, *p* < .001, *d* = 0.87		*t*(47) = 2.97, *p* = .005, *d* = 0.43
Neutral versus unacceptable		*t*(47) = 25.06, *p* < .001, *d* = 3.62	*t*(47) = 7.07, *p* < .001, *d* = 1.02		*t*(47) = 4.73, *p* < .001, *d* = 0.68

The first rows are the statistics for the 3 × 2 ANOVA for each region. In case of a significant interaction, separate ANOVAs with sentence type as a repeated-measure were run. If these were significant, the three sentence types were compared with paired *t* tests

For the trigger position, the interaction was significant and differences in reading times were observed for both trigger conditions, though in different directions. For the determiner, trigger sentences had the longest reading times, while those for neutral and unacceptable sentences did not differ. In contrast, for *again*, reading times were longest for neutral sentences, intermediate for trigger sentences, and shortest for unacceptable sentences.

Also for the post-trigger position, the interaction was significant and differences in reading times were observed for both triggers. For the determiner, differences were small in size, but reading times were longest for unacceptable sentences, intermediate for neutral sentences, and shortest for trigger sentences. For *again*, reading times were longest for unacceptable sentences, but similar for trigger and neutral sentences.

No differences in reading times between the sentence types were obtained for the final word. When considering the total reading time though, reading times depended on sentence type only for *again*, and were longest for neutral sentences, intermediate for trigger sentences, and shortest for unacceptable sentences.

### Discussion

Experiment 1 was largely built on Experiment 1 of Tiemann et al. (2011), however, we focused on the definite determiner and *again* to allow for separate analyses of reading times if warranted. The rating data replicate the results of Tiemann et al. in general, with minor exceptions: Unacceptable sentences were rated worst, and for the trigger *again*, neutral sentences were rated slightly better than trigger sentences. In contrast to Tiemann et al.’s study, trigger sentences were rated better than neutral sentences for the definite determiner. In the original study this was reversed although it is unclear from the report whether the contrasts between sentence types were significant. Overall, this supports the original idea of Tiemann et al. that using presuppositions in neutral contexts is not as unacceptable as using grammatically deviant structures. As a result, successful context integration (i.e., accommodation of the presupposition) should be distinguished from semantic violations.

That context integration did play a role, that is, that participants accommodated the presupposition, is supported by the ratings for the trigger condition, which are unexpectedly quite high, and higher than in the original study. Although the presupposition was not actually mentioned in the context, participants easily accepted the sentences. This suggest that a process of accommodation took place, which was facilitated by the contexts we used. The deviation from Tiemann et al.’s results can be explained by assuming that the contexts used in the present study made accommodation more likely. The observed difference between *again* and the determiner is rooted in the fact that the presuppositions of determiners in general seem to be easier to accommodate (Tiemann et al. 2015).^3^ Based on the ratings, we selected the 32 items that fit our requirements best for use in Experiment 2, namely those sentences that revealed the general pattern we expected most clearly (ungrammatical sentences are worse than trigger sentences which are [slighlty] worse than acceptable sentences).

Reading time results are largely in line with Tiemann et al.’s (2011) observations, but also extend them in an important way. Most importantly, we were able to replicate immediate effects on the trigger and the word following the trigger, with a descriptive pattern very similar to the original study. These results speak for an immediate processing of the presupposition trigger. However, one purpose of the present study was to analyze both triggers separately. While for both trigger types reading times for the trigger positions were longer for trigger than for unacceptable sentences, neutral sentences had the longest reading times for the trigger *again*, but for the determiner, they were similar to those of unacceptable sentences. The long reading times for the neutral condition for the trigger *again* might be due to the unexpected appearance of the word *heute* (Engl. *today*) in this position. It sounds more natural to place the word *heute* at the beginning of the sentence in German. This unexpected word order might have caused the long reading times.

In sum, Experiment 1 replicated effects already on the trigger position for both trigger types, despite our change of presenting all pre-trigger position words simultaneously and testing all items in one session.

## Experiment 2

By and large, Experiment 1 replicated and extended the results obtained by Tiemann et al. (2011). Based on this, Experiment 2 embeds the self-paced reading task within a PRP experiment to apply the locus of slack-logic. The goal is to evaluate whether the processing initiated by a presupposition trigger is (a) automatic and running in parallel with other tasks or is (b) capacity limited with a locus within the central stage of processing. Thus, a binary tone discrimination was added to the self-paced reading task. More precisely, a tone was played after participants read all pre-trigger position words and participants were to respond with a key-press with their left hand to the pitch of the tone. After a variable SOA, the word on the trigger position appeared, and participants proceeded through the remaining sentence in a similar way as in Experiment 1. In terms of the PRP logic (see Introduction), the tone discrimination task can be considered as Task 1, and reading the word on the trigger position would be Task 2.

**Fig. 5:**
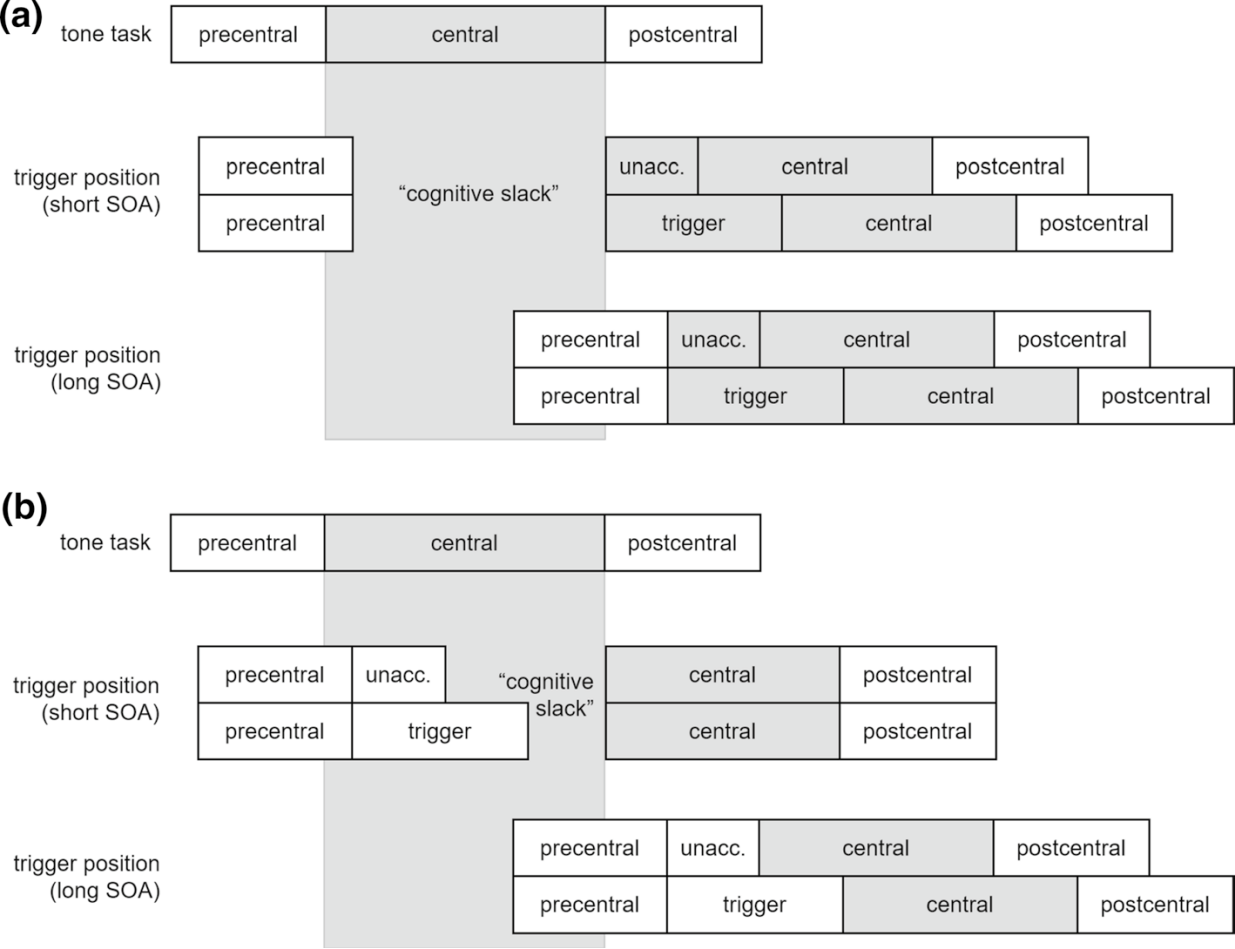
Predictions for reading times at the trigger position for the comparison trigger versus unacceptable (unacc.) sentences. **a** Processing at the trigger position requires central capacity and can thus only start once the central stage of the (preceding) tone task has finished. **b** Processing at the trigger position can run in parallel to other capacity-limited stages and with a short SOA it extends into the cognitive slack. (*SOA* stimulus onset asynchrony)

Because the locus of slack-logic can—in the present setup—only be applied to the word on the trigger position, the predictions for Experiment 2 focus on this position.^4^ We illustrate the predictions for the comparison between trigger and unacceptable sentences in Fig. 5, with the former sentences having resulted in longer reading times for both triggers in Experiment 1. Regardless of whether trigger processing is capacity-limited (Fig. 5a) or automatic (Fig. 5b), differences in reading times for the trigger position are expected with a long SOA. Ideally, the pattern observed there should be the same as already obtained in Experiment 1. Different predictions, however, can be made for the situation with a short SOA. If processing at the trigger position does require central capacity (Fig. 5a), it cannot be initiated before the central stage of Task 1 has finished. In this case, the same differences as with the long SOA are observed and—statistically—sentence type and SOA should combine additively. If, in contrast, this processing is automatic and runs in parallel to the central stage of Task 1, all differences become absorbed into the cognitive slack and should become unobservable with the short SOA (Fig. 5b). Statistically, sentence type and SOA should yield an (underadditive) interaction. In any case, reading times are expected to be longer with a short than with a long SOA, that is, a PRP effect, because some central processing can be assumed anyway, for example, response selection required for pressing the response key (see also Janczyk 2017, for an example).

### Method

#### Participants

The intended sample size in this experiment was *n* = 48. Data were collected from 51 native speakers of German from the Tübingen (Germany) area, of which three participants were excluded because of 30% or more errors in the comprehension questions at the end of the experiment (final sample: mean age = 24.2 years, 39 females, 9 males). Participants signed informed consent prior to data collection and were paid 8€ or received course credit for participation.

#### Apparatus and Stimuli

The same general setup as in Experiment 1 was used. Due to the addition of the auditory (tone) discrimination task, two additional response keys were placed to the left of the participants which were operated with the left index- and middle-finger. Stimuli in this task were 300 and 900 Hz sinusoidal tones of 50 ms length presented via headphones.

Based on the results of Experiment 1, we selected the 32 sets of experimental items per trigger (out of the 50 experimental sets used in Experiment 1) that best fit our expectations regarding the ratings (see “Appendix” for more details on the selection).

#### Task and Procedure

**Fig. 6:**
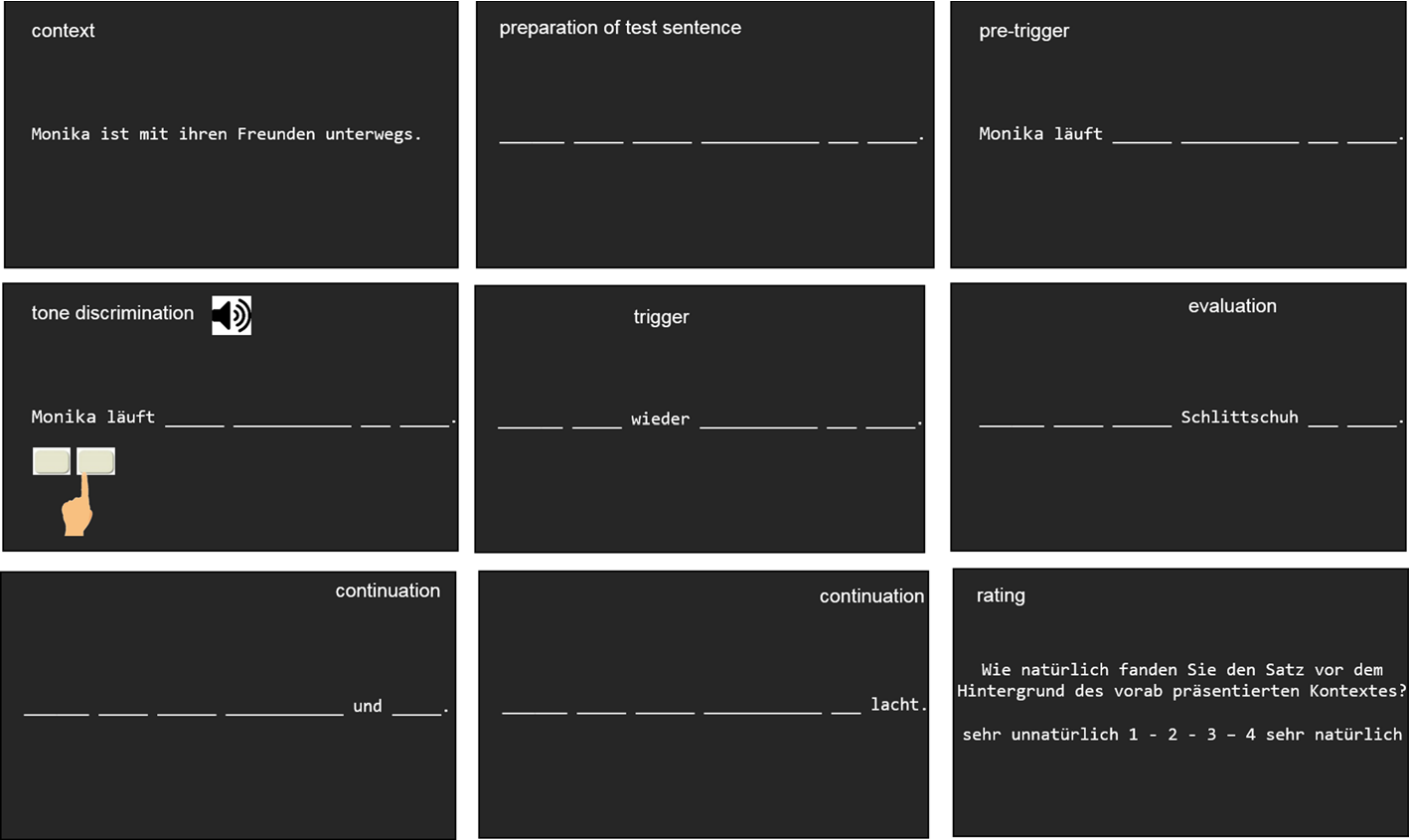
Illustration of the task used in Experiment 2 (see text for more information). (Note that the words appearing in the upper part (“context”, “preparation of test sentence”, …) and the pictures illustrating the tone discrimination task did not actually appear during the experiment but were added here for clarity)

While the general procedure of the self-paced reading task and the acceptability rating was similar to Experiment 1 (see Fig. 6), several changes were required to integrate the auditory discrimination task. To minimize exclusion of trials due to errors in this task, participants started with 40 practice trials of only the auditory discrimination task. Each of these trials was initiated by written instructions centered on the screen that asked the participant to press the right response key to start a trial (to mimic the procedure required in the main experiment). After 100 ms, the 300 or 900 Hz tone was played (each 20 times in a random order) and participants were to respond with a key press of the left hand. In case of errors, respective error feedback was presented on the screen (1000 ms) and the next trial started after an inter-trial interval of 1000 ms.

Then the main experiment began and, similarly to Experiment 1, participants read a context sentence at the beginning of each trial. Pressing the right response key made the context sentence disappear and the underscores appeared as placeholders. With the next key press, all pre-trigger position words appeared. Then, following the next press of the right response key, the 300 or 900 Hz tone was played and following an SOA of 100 or 1200 ms, the next word (on the trigger position) occurred. Participants were to first respond to the tone and then to continue reading. If the response to the tone was correct and no further errors occurred (wrong response order, no response within 5000 ms), the trial was continued until the end of the sentence. As in Experiment 1, this procedure was followed by an acceptability rating task, but in case of errors, the trial was aborted and respective error feedback was presented on the screen (1000 ms).

Participants began with 48 (unanalyzed) practice trials. The 32 (sets) × 2 (trigger types) × 3 (sentence type) = 192 test trials were divided into three test blocks of 64 test sentences each, with a randomized presentation order. The two SOAs were orthogonally distributed across the six combinations of trigger types and sentence types. The stimulus–response mapping of the auditory discrimination task was counterbalanced across participants.

#### Design and Analyses

For all analyses (except on error rates in the discrimination task), trials with an erroneous response in the auditory discrimination task were excluded first (those trials were aborted during the experiment and not continued). Acceptability ratings were analyzed as for Experiment 1. Mean discrimination response times, error rates in the discrimination task, and reading times (per letter) at the trigger position were submitted to a 2 × 3 × 2 ANOVA with SOA (100 vs. 1200 ms), sentence type (trigger vs. neutral vs. unacceptable), and trigger type (determiner vs. again) as repeated-measures.

Trials with response times or reading times deviating more than 2.5 standard deviations from the mean of the respective design cell (calculated separately for each participant) were excluded as outliers.

### Results

First of all, we excluded 2.7% trials with unspecific errors (too slow response, responding to the reading task before responding to the discrimination task, no response within the time limit).

#### Acceptability Rating

Results of the acceptability rating are visualized in Fig. 3b. For the determiner condition, trigger and neutral sentences were rated almost equally well, while unacceptable sentences were rated worst. For *again*, neutral sentences were rated better than trigger sentences, while unacceptable sentences were also rated worst. The ANOVA revealed a main effect of sentence type, *F*(2,94) = 551.27, *p* < .001, η_p_^2^ = .92, ε = .63, but not of trigger type, *F*(1,47) = 0.29, *p* = .595, η_p_^2^ = .01. The interaction was also significant, *F*(2,94) = 4.27, *p* = .025, η_p_^2^ = .08, ε = .79, and we thus analyzed the two triggers separately.

For the determiner, we observed a main effect of sentence type, *F*(2,94) = 478.90, *p* < .001, η_p_^2^ = .91, ε = .59. There was no significant difference between trigger and neutral sentences, *t*(47) = 1.40, *p* = .167, *d *= 0.20, but ratings for unacceptable sentences differed significantly from both the trigger sentences, *t*(47) = 22.65, *p* < .001, *d* = 3.27, and the neutral sentences, *t*(47) = 22.30, *p* < .001, *d *= 3.22. For *again*, the main effect of sentence type was also significant *F*(2,94) = 331.67, *p* < .001, η_p_^2^ = .88., ε = .88, and all differences were significant, trigger versus neutral: *t*(47) = 2.88, *p* = .006, *d* = 0.42; trigger versus unacceptable: *t*(47) = 18.49, *p* < .001, *d* = 2.67; neutral versus unacceptable: *t*(47) = 22.94, *p* < .001, *d* = 3.31.

#### Auditory Discrimination Task

Response times and error percentages are summarized in Table 2 (2.55% outliers). Participants responded more slowly with a short compared to a long SOA, *F*(1,47) = 47.57, *p* < .001, η_p_^2^ = .50, and overall descriptively slightly shorter in the *again* condition, *F*(1,47) = 2.46, *p* = .123, η_p_^2^ = .05. There was also a main effect of sentence type, with slowest responses for unacceptable sentences, intermediate for neutral sentences, and fastest responses for the trigger sentences, *F*(2,94) = 5.02, *p* = .013, η_p_^2^ = .10, ε = .84. This effect was much larger at the short than at the long SOA, thus an overadditive interaction, *F*(2,94) = 9.87, *p* = .001, η_p_^2^ = .17, ε = .77, and for *again* compared to the determiner, *F*(2,94) = 4.20, *p* = .018, η_p_^2^ = .08. There was no significant interaction of SOA and trigger type, *F*(1,47) = 0.40, *p* = .532, η_p_^2^ = .01. Because the three-way interaction was significant, however, *F*(2,94) = 3.72, *p* = .028, η_p_^2^ = .07, separate 3 × 2 ANOVAs were run for each trigger type.

**Table 2:** Mean response times (in milliseconds)|error percentages for the auditory discrimination task as a function of sentence type, trigger type, and stimulus onset asynchrony (SOA)

Sentence type	Trigger type			
	Determiner		Again	
	SOA (ms)		SOA (ms)	
	100	1200	100	1200
Trigger	823|3.63	763|1.70	801|2.49	734|1.83
Acceptable	843|1.98	752|2.25	810|2.41	751|2.15
Unacceptable	860|3.29	743|1.97	891|1.60	732|2.92

For the determiner, only the main effect of SOA was significant, *F*(1,47) = 42.72, *p* < .001, η_p_^2^ = .48. Neither the main effect of sentence type, *F*(2,94) = 0.33, *p* = .722, η_p_^2^ = .01, nor the interaction were significant, *F*(2,94) = 3.04, *p* = .059, η_p_^2^ = .06, ε = .89. For *again*, both main effects were significant, SOA: *F*(1,47) = 40.15, *p* < .001, η_p_^2^ = .46; sentence type: *F*(2,94) = 9.28, *p* < .001, η_p_^2^ = .16, as was the interaction, *F*(2,94) = 12.53, *p* < .001, η_p_^2^ = .21, ε = .84.

For error rates, no effect reached significance, all *F*s ≤ 3.06, all *p*s ≥ .087.

#### Self-Paced Reading Task

**Fig. 7:**
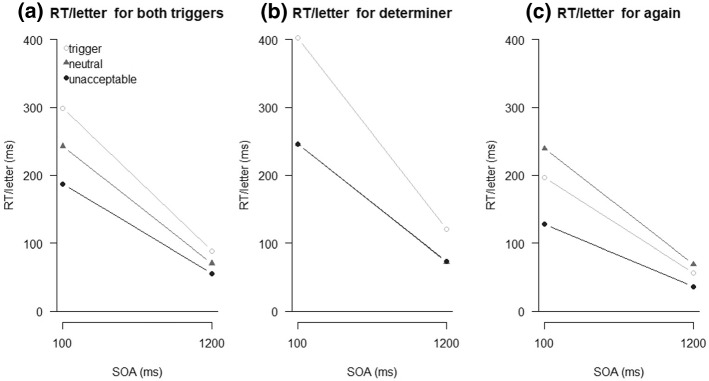
Reading times (RT) for the trigger position as a function of sentence type and stimulus onset asynchrony (SOA) in Experiment 2 analyzed across triggers in (**a**), and separately for the two triggers determiner (**b**) and *again* (**c**)

Mean reading times (per letter) for the trigger position across both trigger types are visualized in Fig. 7a (2.77% outliers). Sentence type influenced reading times significantly, *F*(2,94) = 471.53, *p* < .001, η_p_^2^ = .91, ε = .76. Furthermore, we observed a main effect of SOA, *F*(1,47) = 1539.54, *p* < .001, η_p_^2^ = .97, with shorter reading times with a long SOA, and a main effect of trigger type, *F*(1,47) = 495.16, *p* < .001, η_p_^2^ = .91. Sentence type and SOA also interacted, *F*(2,94) = 231.57, *p* < .001, η_p_^2^ = .83, but the effect of sentence type was *larger* with a short than with a long SOA, that is, the interaction was *over*additive. Sentence type and trigger type also interacted, *F*(2,94) = 376.94, *p* < .001, η_p_^2^ = .89, ε = .67, and the interaction of SOA and trigger type was significant as well, *F*(1,47) = 717.47, *p* < .001, η_p_^2^ = .94. Finally, the three-way interaction was also significant, *F*(2,94) = 175.37, *p* < .001, η_p_^2^ = .79, ε = .84, and we thus analyzed both trigger types separately.

For the trigger determiner (Fig. 7b), the main effect of sentence type was significant, *F*(2,94) = 391.44, *p* < .001, η_p_^2^ = .89, ε = .71, as was the main effect of SOA, *F*(1,47) = 1590.31, *p* < .001, η_p_^2^ = .97. Further, we observed a significant interaction of sentence type and SOA, *F*(2,94) = 186.88, *p* < .001, η_p_^2^ = .80, ε = .82. A very similar picture was obtained for the trigger *again* (Fig. 7c). The main effect of sentence type was significant, *F*(2,94) = 542.56, *p* < .001, η_p_^2^ = .92, ε = .78, as was the main effect of SOA, *F*(1,47) = 1224.45, *p* < .001, η_p_^2^ = .96. The interaction of sentence type and SOA was also significant, *F*(2,94) = 259.45, *p* < .001, η_p_^2^ = .85.

### Discussion

Experiment 2 investigated whether the immediate processing induced by presupposition triggers is automatic or requires limited capacity. To this end, the PRP approach and the logic of slack-logic were combined with the self-paced reading task already used in Experiment 1.

The ratings largely replicated the results obtained in Experiment 1 with the exception that the difference between neutral and trigger sentences was not significant for the determiner. The overall higher ratings for trigger sentences compared to unacceptable sentences suggest that participants accommodate the presupposition and interpret the sentences (and presuppositions) as intended.

Regarding the tone discrimination task, the main effect of SOA is somewhat unexpected against the background of the central bottleneck model. However, such observations are not uncommon in PRP research. We will come back to this in the General Discussion, where we also consider the overadditive interaction of sentence type and SOA.

Regarding reading times on the trigger position, we obtained several results of interest. First, with a long SOA, the observed pattern of differences between sentence types was the same as in Experiment 1, and thus a successful replication of these results. Secondly, these differences were the same with a short SOA. This result contradicts the notion that the processes initiated by the trigger are automatic. It is noteworthy though that the differences were even larger with a short than with a long SOA—a pattern that is also not predicted by the underlying central bottleneck model. We will come back to this in the General Discussion.

## General Discussion

The purpose of this study was to investigate the nature of the immediate processing initiated upon encountering a presupposition trigger. To that end, we compared sentences including a presupposition trigger with grammatical sentences without a presupposition trigger and unacceptable sentences. Experiment 1 replicated several results obtained by Tiemann et al. (2011), but focused only on two triggers, namely definite determiners and *again*. Experiment 2 combined a self-paced reading task with the PRP approach and the locus of slack-logic to address the main question of the present study: Is presupposition processing an automatic or capacity-limited process?

### Main Results and Theoretical Implications

Experiment 1 replicated the immediate effects of presupposition processing on the presupposition trigger, as observed by Tiemann et al. (2011). Furthermore, our first experiment revealed that presenting all words preceding the trigger simultaneously and presenting all relevant stimuli in one test session did not influence the immediate effects on the trigger itself or the participants’ interpretation.

Experiment 2 was based on the results of Experiment 1 and assessed whether the observed processing initiated by the presupposition trigger is automatic or capacity-limited. The former leads to the prediction that the trigger effects should only be observed with a long but not with a short SOA. This was clearly not the case, and thus an automatic processing of presupposition triggers appears unlikely. Admittedly, the observed overadditive interaction (i.e., a larger trigger effect [of sentence type] with the short than with the long SOA) is also not predicted by the central bottleneck model.^5^ However, we can tentatively offer an explanation for this result. In particular, a very similar pattern was observed for the response times in the tone discrimination task. With a short SOA, differences in this task “propagate” into the reading times of the subsequent task, in this case the reading times at the trigger position. This might have induced the overadditivity we observed. Although this is a post hoc explanation, it seems important to again point out that the observed results are certainly not compatible with automatic trigger processing.

The findings are in line with assuming that for presupposition triggers a context search is started immediately: For the definite determiner, the search for an appropriate referent is started, and for *again* a suitable previous event has to be found in the context. It is important to note that this search for a referent must initially be unsuccessful in the presented context, as the presupposition was never explicitly verified, that is, a clear referent for the definite noun phrase was never given and there was no previous event that *again* could refer to either. As a result, participants possibly anticipated to accommodate the presupposition, which the acceptability ratings suggest they did. Accommodation is a poorly understood process, both from the theoretical and experimental point of view. However, under any theory it is usually assumed to be a process of enriching the context with the information of the presupposition, if that is contextually feasible. To check whether adding the presupposition is plausible requires good knowledge of the contents of the context. It is thus unsurprising that in preparation of accommodation cognitive resources like working memory play a role already on the trigger itself.

From a different perspective, assuming a search process is well in line with previous research suggesting a link between presupposition processing and working memory. For example, Anderson and Holcomb (2005) compared test sentences that either included a definite or an indefinite determiner (e.g., “The/A cab came very close to hitting a car.”) with a context sentence preceding these test sentences that either introduced the critical noun directly or used a synonym (e.g., “Kathy sat nervously in her cab/taxi to the airport.”). The data revealed an enhanced left anterior negativity (LAN) for the definite compared with the indefinite determiner, reflecting a referential assignment of the noun phrase following the definite determiner to an antecedent. King and Kutas (1995) also interpreted the increase in the LAN as a referential assignment that increases the demands on working memory (see also Domaneschi et al. 2014). Conceivably, this requires encoding of information into working memory, and exactly this has been shown capacity-limited (Jolicoeur and Dell’Acqua 1998). Furthermore, this assignment is only possible when the relevant entity is found in memory. The necessary search process in turn likely involves repeated selection and de-selection of working memory items that are considered as referents, and selecting working memory items is also a capacity limited process (Janczyk 2017).

Regarding potential similarities and differences in processing between both triggers, the results suggest that both triggers share the feature of capacity-limitations. As suggested, we suspect that the underlying process requiring cognitive capacity is indeed the same. At the same time, however, the numerical difference of the size of the interaction between sentence type and SOA may point to differences. These could result from different difficulties in the underlying search for a potential referent or from an additional process running only for one of the triggers. The present data do not allow for drawing definite conclusions on this matter, however. Moreover, based on our data, a direct comparison between the two triggers is not feasible as we did not control for several factors influencing differences in processing, for example, syntactic position. The present paper offers an indirect comparison by showing that processing both presupposition triggers is capacity-limited. They may require different amounts of capacity and/or different processes; however, the qualitative conclusion is the same for both investigated triggers.

We would like to make two additional comments regarding a comparison. First, a direct comparison between triggers, and especially the one between the definite determiner and *again,* is hardly ever possible, even if many factors are controlled for. This is because both words belong to different categories (determiner vs. adverb), and, as a result, necessarily appear in different syntactic positions and fulfill different syntactic roles. They also occur with different frequency. Second, we believe we did control for several factors that were relevant for our critical Experiment 2. In particular, we tested for naturalness, readability, and predictability by including the acceptability rating task, which should reveal any deviances in these respects. The findings reveal consistent behavior of participants in judging the sentence with the trigger to be acceptable. Further, the role of position was reduced in our experiment as we presented the part before the trigger position simultaneously for all sentence types. In other words, the critical word of investigation always occurred in the second position. Finally, the total word length of the sentence could not have influenced interpretation of the word at the trigger position and, in the critical Experiment 2, this is the only position of interest for the locus of slack-logic.

### Limitations and Future Extensions

The probably clearest limitation of the present study is its focus on only two particular triggers in only one language (German). One reason for this choice was to increase the amount of test sentences and thereby improve the precision of aggregate estimates compared to the original study by Tiemann et al. (2011). We purposefully chose triggers belonging to different classes, that is, *again* as a representative of Class 1 and definite determiners as a Class 2 member according to the classification by Tiemann et al. (2015).^6^ However, a generalization on the basis of only these two triggers is impossible, and consequently, additional data are needed to gain further insights into potential classes of triggers. Furthermore, future studies should be carried out in different languages than German, to explore the generality of our results across different languages. The important contribution of the present paper is that the methodology and logic of our experiment can be fruitfully extended to investigate difference between (other) triggers and non-triggers, as well as different languages.

Another objection may relate to the choice of the central bottleneck model from which we derived the predictions for Experiment 2. It is certainly true that the adequacy of this model is debated in cognitive psychology. Rather, models have been suggested that allow for parallel processing of the central stage as well, even though the assumption of a capacity-limitation is still made (Navon and Miller 2002; Tombu and Jolicoeur 2003).While parallel processing is possible, the available capacity must be divided between tasks leading to performance decrements in turn. The exact proportion is, for example, determined by a sharing parameter in the model of Tombu and Jolicoeur: if all capacity is first devoted to Task 1, their model mimics the central bottleneck model. The important point here is, however, that the critical predictions for Task 2 (in our case: reading the word at the trigger position) are the same for capacity sharing models and the central bottleneck model. It is simpler, though, to derive and illustrate the predictions from the latter model. Regarding Task 1 performance (in our case: the tone discrimination task), both types of models indeed differ in their predictions. However, the effect of SOA and the overadditive interaction of sentence type and SOA in response times in the auditory discrimination task are more compatible with the capacity sharing approach.

## Conclusion

The present study replicates and extends previous results regarding immediate processing of presuppositions, starting on the respective triggers. Two main conclusions can be drawn based on the two experiments we reported: First, encountering a presupposition trigger indeed appears to induce immediate processing of the presupposition. Second, this processing requires limited cognitive capacities and is not automatic.

## Figures and Tables

**Fig. 8:**
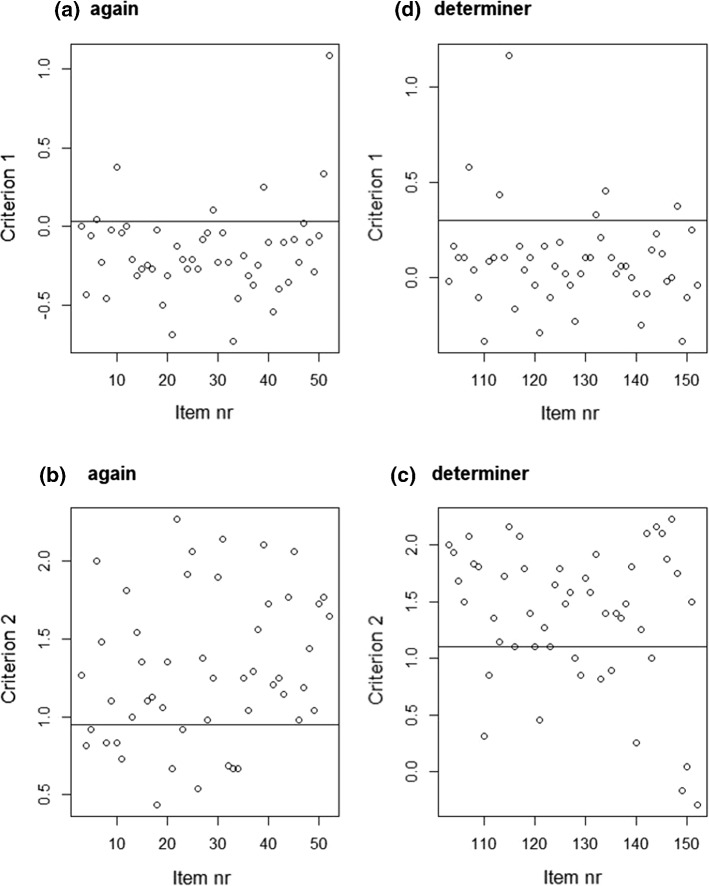
Visualization of the differences between the relevant sentence types for *again* (**a**, **b**) and determiner (**c**, **d**) that were used to select the 32 items for Experiment 2. The exclusion criteria are visualized with the black line

## Appendix

The items used in Experiment 2 were selected on the basis of the ratings obtained in Experiment 1 with regard to two criterions. Criterion 1 was that the difference between the ratings of neutral sentences and trigger sentences was minimal (and at best: negative), and Criterion 2 was that the difference between trigger sentences and unacceptable sentences was maximal. The exact exclusion criteria for the two trigger types were set in a way that approximately one third of the trials were excluded for Criterion 1, and two thirds were excluded due to Criterion 2. For the trigger *again*, exclusion criteria were a value > 0.03 (Criterion 1) or a value < 0.095 (Criterion 2). For the trigger determiner, exclusion criteria were a value > 0.25 (Criterion 1) or a value < 1.00 (Criterion 2) (see Fig. 8 for a visual illustration). The six items for each trigger that were closest to the exclusion criteria were used for the practice block.

## Footnotes

1 We are simplifying a bit here, as these accounts do not necessarily make these claims for all presupposition triggers but make further distinctions. As the direct comparison of these different classes of triggers is not the main focus of our paper, we will not go into the details of this part of the debate.

2 In this experiment, the authors actually compared performance in a PWI task with performance in a color word Stroop task. For simplicity, we here only consider the PWI task.

3 We would like to point out though that we can only draw weak conclusions about accommodability based on our data, as we did not explicitly control for factors that have been shown to influence the availability of accommodation, for example, plausibility, bridging etc. It is important to note, however, that this is not the main point of the present study, but rather the comparison of presuppositional sentences to non-presuppositional ones, both grammatical and ungrammatical, to test whether processing presuppositions is capacity limited.

4 In principle, of course, the PRP and locus of slack-methods can be applied to other positions as well. Yet, one has to conduct a separate experiment, because with the locus of slack-logic it is only possible to investigate one position at a time. Thus, a similar investigation, for example, for the point where the presupposition becomes completely known cannot be done within the same experiment and must be postponed to future research.

5 The overadditive interaction remained significant when the neutral sentences were excluded, and thus was not driven by these sentences only.

6 There exist other classifications of triggers with which our results are also in line with, for example, informative versus non-informative (Tonhauser 2015), soft versus hard (Abusch 2010), and entailed versus non-entailed (Klinedinst 2016; Sudo 2012).
